# Perturbing the Dynamics and Organization of Cell Membrane Components: A New Paradigm for Cancer-Targeted Therapies

**DOI:** 10.3390/ijms19123871

**Published:** 2018-12-04

**Authors:** Nuno Bernardes, Arsenio M. Fialho

**Affiliations:** 1 iBB-Institute for Bioengineering and Biosciences, Biological Sciences Research Group, Av. Rovisco Pais 1, 1049-001 Lisbon, Portugal; 2 Department of Bioengineering, Instituto Superior Técnico, University of Lisbon, 1049-001 Lisbon, Portugal

**Keywords:** membrane targeted therapy, membrane rafts, cancer therapy, azurin, membrane modulation

## Abstract

Cancer is a multi-process disease where different mechanisms exist in parallel to ensure cell survival and constant adaptation to the extracellular environment. To adapt rapidly, cancer cells re-arrange their plasma membranes to sustain proliferation, avoid apoptosis and resist anticancer drugs. In this review, we discuss novel approaches based on the modifications and manipulations that new classes of molecules can exert in the plasma membrane lateral organization and order of cancer cells, affecting growth factor signaling, invasiveness, and drug resistance. Furthermore, we present azurin, an anticancer protein from bacterial origin, as a new approach in the development of therapeutic strategies that target the cell membrane to improve the existing standard therapies.

## 1. Introduction

The role played by the plasma membrane and its biophysical status in cancer is increasingly being recognized in the field of anticancer therapies. Usually, genetic alterations are the trigger for oncogenic transformation, which in many cases leads to changes in the lipid profile and biophysical properties of cells, impacting the response to the various conventional therapies [1]. Most anticancer drugs act on membrane surface receptors or have intracellular targets (e.g., DNA) needing to interact and/or cross cellular membranes to achieve their target sites. Hence, membranes act as additional barriers upon malignant transformation promoting lipid and/or protein–drug interactions and modulating their efficacy. Limited efficacy of the drugs can have as consequence the development of the multidrug resistance (MDR) phenomenon and development of metastasis [2,3]. Multiple factors contribute to MDR such as the heterogeneity of the population which can cause intrinsic resistance in some clonal populations over others, but resistance can also be acquired. The result is often the same, i.e., the reduction in the effective concentration of drug in the cell before it reaches its cellular target. Among others, the mechanisms reported comprise overexpression of drug efflux pumps; alterations in lipid metabolism (ceramide–Cer–pathway); elimination of the drugs by cellular detoxification systems; sequestration of drugs inside lysosomes and endosomes; reduced drug uptake caused by altered surface receptors (G-protein family and/or solute carries); or altered signal transduction pathways regulated by cell surface receptors such as growth factor receptors and/or integrin receptors (for a more extended review see [2]).

Another feature adding to these is the reduced free diffusion of anticancer drugs through the plasma membrane [1]. Therefore, studying the alterations occurring at membranes during cancer progression is a growing field of research which may impact the design of new drugs and therapeutic approaches in the near future. In fact, several anticancer drugs are being evaluated based on their capacity to exert therapeutic effect by modulating the properties of tumor membranes, thus being the basis for membrane-lipid therapies [4,5,6,7].

## 2. Membrane Biophysics of Cancer Cells

The rapidly dividing nature of cancer cells creates a need for altered biosynthetic pathways and the synthesis of fatty acids is frequently up-regulated in these cells to form new membranes [8,9,10]. However, the biophysical constraints imposed by the nature of the lipids present in the membranes frequently produce alterations in cancer cells, although the nature of the changes differs between different tumors types, stage or sensitivity to drugs [11,12].

Lipid molecules comprise a polar head and a hydrophobic tail with a relatively long hydrocarbon chain length. The thermodynamic forces regulating the behavior of these molecules give rise to a spontaneous association in structures such as micelles or bilayered sheets which protects the hydrophobic tails surrounded by the hydrophilic heads [6,13]. The differences in the lipid polar heads groups or the hydrocarbon chain then results in different properties such as overall structure, charge, packing, and thus, the behavior of the membrane. For example, phophatidylcholine (PC) and sphingomyelins (SM) have a cylindrical shape. Due to its large polar head and amphiphilicity, PC usually forms a lipid bilayer in a water-environment. On the contrary, most phosphatidylethanolamine (PE) lipids are considered cone-shaped lipids and do not form lipid bilayers by themselves, a property that is dependent on the length and level of unsaturation of the acyl chains, which is critical for the embedding of membrane proteins. Poliphosphoinositides (PI) however, due to the higher head to tail proportion have an inverted cone-shape form, causing positive membrane curvature. Sphingolipids contain very long fatty acid chains forming solid gel phases fluidized by sterols. Therefore, changes in the lipid composition strongly affect the overall behavior of the membrane [6,14].

**Figure 1:**
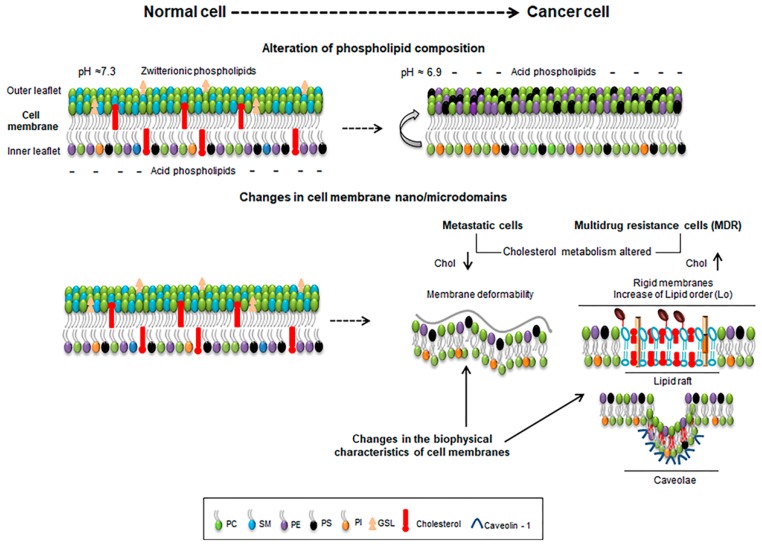
Schematic representation of some features of normal and cancer cells depicting lipid composition and extracellular pH (upper panel), and the alterations occurring at the cellular membrane nano/microdomains (lower panel). Metastatic cells with lower cholesterol levels (chol ↓) enhance membrane deformability (lower panel, left) while cells with increased cholesterol levels (chol ↑) enrich membrane in ordered lipid raft domains and rigidity (lower panel, right).

Membranes of normal cells are characterized by a lipid asymmetry between the inner and outer leaflets. Zwittterioninc lipids such as PC and SM are prevalent in the outer leaflet while phospholipids containing amine (phosphatidylethanolamine) (PE) or serine (PS) are more represented in the inner leaflet (Figure 1). This asymmetry is maintained by enzymes such as flipases, scramblases and translocases being an active and energy-dependent process [6,15]. The negative surface charge due to the prevalence of aninonic PS in the inner leaflet influences numerous signaling mechanisms such as the hydrolysis of signaling lipids such as phosphatidylinositol (PI) by phospholipase C into inositol 1,4,5-triphosphate (IP3) and Diacylglycerol (DAG) that act as secondary messengers. Also, the enrichment of PE and PS in the inner leaflet stabilizes interactions with the cytoskeleton by the amine and serine moieties, forming a fence that limits the free lipid movement within the membrane and contributes to its curvature and mechanical properties [16]. A de-regulation of this asymmetry is often encountered in pathological conditions such as cancer in which cells often expose PS in the outer membrane resulting in a negative surface charge of cancer cells, which also correlates with a more acidic pH of their external media [17,18]. In tumor endothelial cells, PE was also found exposed in the outer leaflet of the cells [19].

The different structure and physical properties of the lipids in the membrane also causes changes in its fluidity. Depending on the temperature, lipids change from gel solid-ordered phase (So) with extended hydrophobic tails, occurring a change at the transition temperature (Tm), where lipids form a liquid-disordered state (Ld), where the hydrocarbon tails are not stretched. At the physiological body temperature, most membranes are at a Ld state due to its lipid composition [20,21], but membrane fluidity is also strongly influenced by cholesterol content and the lipid unsaturation degree [22,23]. Cholesterol (Cho) is a major component of plasma membranes. When its content is between 8–15% membranes remain in the Ld state, although at different possible degrees. Increasing values of cholesterol (20–40%), however, cause a change to a more rigid liquid-ordered (Lo) membrane state [6,24]. Both membrane states co-exist in plasma membranes in which we can found areas with an enrichment in certain lipids, forming domains. This mode of organization of the membranes is essential for its role in several cellular processes such as polarization, trafficking, and endocytosis, and mechanotransduction of external cues to intracellular signaling. The main lipid domains found in plasma membranes are *caveolae* and lipid rafts [25]. Lipid rafts are nanodomains located on the outer leaflet of the membranes, enriched in sphingolipids (SL) and cholesterol [13,26]. Their assembly and dynamics occur very rapidly through van der Waals forces and hydrogen bonds between the OH- group of cholesterol and the SL [27,28]. These membrane domains are associated with the Lo phase of the membrane providing a platform for certain proteins [6,29] (Figure 1).

Cer-enriched lipid rafts are micro or macrodomains in size. The small polar head of Cer promotes a self-association stabilized by intermolecular hydrogen bonds, forming stable and highly ordered domains [30]. The presence of even small amounts of Cer is sufficient to displace cholesterol from lipid raft Lo nanodomains which improves the overall order of the membrane [6]. Combined with a higher lifetime than Lo nanodomains and lower lateral mobility, Cer lipid rafts establish longer associations to embedded proteins. To clarify the role of these two lipid raft domains, some authors have proposed the denomination of the two different lipid rafts, being Type 1 the cholesterol and shingolipid-enriched domains and Type 2 the Cer-enriched domains [6,31,32]. Both domains are considered to be hot spots for signaling since important proteins are associated with them through different post-transcriptional modifications [13,25,33,34,35]. GPI anchored proteins [13,36], growth factor receptors such as EGFR (epidermal growth factor receptor) [37,38] and IGF-1 (insulin-like receptor) [12] are proteins known to associate to Type 1 lipid rafts, while proteins involved in apoptosis, such as Fas (CD95), TNF-R1 or TRAIL receptor, are associated with Type 2 lipid rafts. The *caveolae* domains are enriched in cholesterol, sphingolipids and lipid-anchored proteins in which the polymerization of the coat protein caveolin-1 is needed to form surface invaginations with 50–100 nm of diameter [39,40].

Cholesterol metabolism is frequently altered in cancer cells resulting in either lower or higher values that may vary according to the type of cancer, stage and sensitivity [1,6,12]. Indeed, in metastatic cells lower cholesterol levels correlate well with a more deformable membrane increasing their ability to enter in blood vessels [41,42]. However, in multidrug resistant cells (MDR), higher values of cholesterol can be found, turning the membrane more rigid and, thus, less permeable for drugs [1] (Figure 1). Besides cholesterol, also PC, PE and PI are more abundant in MDR cells than normal cells. The increase in cholesterol promotes the presence of Lo lipid raft domains contributing to malignant transformations, hyper growth and invasiveness [6,12]. Also, in general a decrease in drug influx correlates to the membrane biophysical properties. Doxorubicin-resistant P338 cell line had a decreased PC/SM ratio and an increase in the membrane order [1,43]; Vinblastine-resistant leukemia T cells showed an increase of up to 60% in protein/lipid content compared to drug-sensitive cells [44]. Peetla et al. reported that lipids isolated from doxorubicin-resistant MCF-7 breast cancer cells (MCF-7/ADR) had higher concentrations of SM, PI, and cholesterol than sensitive cells and that membrane lipids are more rigid than sensitive cells, showing also that doxorubicin had strong hydrophobic interactions with resistant cell membrane lipids when compared to lower and ionic interactions that established with membrane lipids from sensitive cells [45].

Apart from the growth factors already referred, Lo lipid rafts also harbor other proteins such as integrins, CD44 and CD24 receptors that are involved in tumor progression [36,46]. In particular, in MDR cells transporters such as P-glycoprotein (P-gp) that have a role in the efflux of chemotherapeutic drugs from the cytoplasm to the extracellular space are located within lipid rafts [47,48,49]. Several studies relate not only the presence of P-gp in lipid rafts but also the direct influence of this membrane environment in its activity (for a more extended review see [1]). Using model membranes, Alves et al. [50] studied the interaction of doxorubicin with lipids to evaluate the impact of this drug in the organization of the plasma membrane. Doxorubicin was found to locate preferentially in ordered domains of the membrane, which could then contribute to its enhanced efflux by P-glycoprotein.

The metabolism of Cer is also an effective drug resistance mechanism used by cells [51,52]. In cells, Cer is an intermediate metabolite of more complex SL or is the result of Sphingomyelinase (SMase) activity (which produces Cer from SM). The activity of SMase produces then a very tight control on Cer levels and controls different processes such as differentiation, proliferation, and apoptosis. Low levels of Cer are found in MDR cells either by lowering the activity of SMase or by increasing the SM levels. Therefore, low levels of Type 2 lipid rafts are formed, preventing apoptosis [46].

As seen, the pathological conditions found in cancer can alter membrane-lipid composition, its biophysical properties and nano/microdomains dynamics with consequences at the intracellular downstream signaling events. Therefore, drugs interaction and regulation of membrane properties can be used to modulate membranes and potentiate new therapeutic strategies.

## 3. Therapeutic Strategies and Anticancer Drugs to Modulate Membranes

Conventional anticancer drugs are mostly designed to interact with proteins or nucleic acids. Currently, drugs that target lipids or affect membrane-lipid composition are becoming interesting alternatives to target the aforementioned changes observed in cancer cells. The modulation of the membrane biophysics or the targeting of the specific lipid alterations changing its composition and structure may alter the domain dynamics and/or the affinity of one or more proteins involved in the transduction of specific signaling [6,53].

### 3.1. Modulation of Invasion and Proliferation Associated Pathways by Lowering Cholesterol Content

The removal of cholesterol from the plasma membranes reduces the prevalence of Lo lipid raft domains causing the dissociation of the proteins there located. The use of chemical agents such as filipin which binds cholesterol preventing its association to SL, or chemical remove using methyl-beta-cyclodextrin (MβCD), results in hampering the propagation of their signaling events. Pathways associated with EGFR or estrogen receptors often overexpressed are severely decreased upon cholesterol depletion [6,12,38].

The main biologically active component of green tea, epigallocatechin gallate (EGCG), inhibits the EGFR activation and its downstream signaling pathways in several human cancer cell lines [12,54,55]. In HT-29 colon cancer cell lines deprived of epidermal growth factor (EGF), it was seen that EGFR is mainly located at in Ld domains but upon activation with EGF or TGFα, EGFR translocates to Lo domains, to produce its downstream signaling. However, EGCG disabled the ordered domains thus preventing the proliferative signaling [56]. The same type of preventive effects was seen in head and neck squamous cell carcinomas (HNSCC) [57]. Additionally, in HNSCC EGCG demonstrated a synergistic effect with erlotinib, an inhibitor of EGFR [58]. Other examples of synergistic effect of EGCG are found in trastuzumab-resistant BT474 breast cancer cell line [59] and tamoxifen-resistant breast carcinoma cells. In both cases, EGCG caused dose-dependent apoptosis facilitating the action of each drug.

Malignant cells depend strongly on the ability to synthesize new fatty acids (FA) to provide the required elevated levels of building blocks for the newly synthesized membranes [60]. For that reason, the levels of the FASN (Fatty acid synthase), an enzyme required for this increased lipogenesis is often overexpressed in malignant cells. FASN catalyzes the condensation of one acetyl-coenzyme A (acetyl-coA) with seven molecules of malonyl-coA originating long-chain FA, in particular palmitate. This 16-carbon saturated FA can be further elongated or desaturated forming other FA by other enzymes such as stearoyl-CoA desaturases (SCDs) [10]. Increased levels of SCDs have also been associated with tumorigenesis and poor prognosis in several cancers. Moreover, it has been shown FASN and caveolin-1 can interact both physically and functionally in prostate cancer cells, suggesting an involvement with membrane ordered domains [61]. However, despite its importance in cancer progression several attempts to pharmacologically target FASN have failed until now, but a new FASN antagonist (TVB-2640) is now under evaluation in Phase I and II clinical trials [62].

Another lipogenic pathway is the mevalonate (MVA) pathway, which begins with acetyl-CoA and produces isoprenoids such as cholesterol [60]. Statins lower cholesterol levels by blocking the conversion of 3-hydroxy-methylglutaryl (HMG) coA to mevalonic acid through the inhibition of the HMG coA reductase enzyme to which statins are structurally similar [63]. Cancer cells often present an upregulation of the mevalonate pathway [63,64], therefore acting on the early stages of this pathway will decrease the lipid raft content and membrane rigidity [6]. Statins not only block the synthesis of cholesterol but also of other metabolites such as the isoprenoids geranylgeranyl pyrophosphate (GGPP) and farnesyl pyrophosphate that activate farnesylated proteins of the Ras family and geranylgeranylated proteins such as RhoA and Rac1 [12,63]. Lovastatin was able to induce apoptosis in ovarian cancer through a mevalonate-dependent mechanism while also displaying synergistic effects with doxorubicin [65]. Another statin, atorvastatin, in combination with carboplatin (a platinum-based anticancer drug) enhanced the survival of lung cancer cells A549-bearing nude mice compared to carboplatin only through the inhibition of AKT [66].

In addition to alterations in the synthesis, the β-oxidation of FA also provides energy to the cells, as ATP, NADPH for the reduction of oxidative stress and acetyl-coA for protein acetylation and both mechanisms play roles in cancer cell maintenance since recently cancer has been shown dependent on both FA synthesis and oxidation [60].

Emodin (3-methyl-1,6,8-trihydroxyanthraquinone) is an active component found in the roots and rhizomes of *Rheum palmatum* L. [12]. This compound was found to have similar effects on cholesterol levels as MβCD, lowering its levels in isolated detergent-resistant membranes (DRM) fractions, as well as of the main SL [67]. Lipid raft-associated events such as translocation of integrin subunit β1 and the formation of focal adhesions were prevented by both emodin and MβCD, an effect that was lost after cholesterol replenishment [67]. In MDA-MB-231 breast cancer cells, emodin also inhibited the co-localization of integrin β1 with lipid rafts [67]; in breast and liver carcinomas it was found to inhibit cell migration by suppressing PI3K-Cdc42/Rac1 signaling [68]; and in lung and prostate carcinoma emodin prevented the invasion and migration by down-regulating the CXCR4 chemokine receptor [69].

### 3.2. Stabilization of Pro-Apoptotic Membrane Domains

As mentioned before, Type 2 lipid rafts are mainly pro-apoptic Cer-enriched membrane domains. Therefore, in this approach, the strategy is to enhance the Cer rafts, displacing cholesterol from Type 1 lipid rafts therefore inducing apoptosis in cancer cells [6]. Synthetic alkylphospholipids (ALPs) are a class of new anticancer drugs chemically divided in two main classes: alkylysophospholipids (ALP) and alkylphosphocolines (APC). These agents target cell membranes by interfering with lipid homeostasis due to their similarity with endogenous phospholipids. ALPs act by modulating membrane permeability and fluidity altering lipid-linking signaling, phospholipid metabolism, proliferation and, ultimately, leading to apoptosis [5,11]. Edelfosine was the first agent of this class reported to induce apoptosis in cancer cells. In multiple myeloma cells, edelfosine demonstrated great ability to induce modifications in membrane-lipid raft structure clustering Fas/CD95 death receptors and lipid rafts [26,70,71,72]; however, its clinical application has been limited due to gastrointestinal toxicity, hemolytic potential, and metabolic instability [5]. Edelfosine analogue miltefosine lacking the glycerol motif was the first ALP used in clinic for the local treatment of cutaneous metastasis of breast cancer, but as the lead compound edelfosine its hemolytic activity prevented its extension to other tumor types [5]. A modification in miltefosine replacing the choline moiety by a heterocyclic pipepridine group resulted in perifosine which demonstrated increased half-life and stability preventing its rapid degradation [73,74]. It also induces apoptosis in carcinoma cell lines [75], recruiting Fas/CD95 to lipid rafts [76]. Despite phase II clinical trials using perifosine as single-agent still demonstrated high levels of gastrointestinal toxicity, it has had clinical success as an AKT inhibitor in combination with other anticancer agents [5]. Indeed, one of the most studied pathway targeted by ALPs is the PI3K/Akt/mTOR pathway, particularly Aky which is frequently overexpressed in many cancer types (for a detailed review see [5]). The ethyl phosphate 10-(Octyloxy) decyl-2-(trimethylammonium) (ODPC) is another ALP that interacts with the cell membrane targeting high cholesterol domains inducing apoptosis in leukemia cells but not having cytotoxic effects in normal hematopoietic cells [77]. Furthermore, in lipid model systems containing phase coexistence of Lo-Ld domains, the presence of ODPC disrupted the domains [78].

The natural polyphenol resveratrol (3,5,4’-trihydroxystilbene) is also capable of inducing apoptosis in a colon cancer cell line by clustering the Fas/CD95 receptors to membrane rafts without ligand activation [79]. It accumulates in lipid rafts before its endocytosis [80] and even for the HT-29 resveratrol-resistant cell line, its pre-treatment enhances greatly apoptosis induced by the natural ligands TNFα, TRAIL or anti-Fas/CD95 antibodies [79].

The equilibrium between Cer and SM is then fundamental for the cells to regulate proliferation and apoptosis. The levels of two enzymes that, among others, regulate the levels of these two lipids, Glucosylceramide synthase (GCS) that converts Cer into SM and SMase that converts SM to Cer phosphate, are frequently de-regulated in cancer cells. Drug resistant cells often overexpress GCS [6,81] or down-regulate SMase [1,52,82,83] to favor proliferation over apoptosis. Several strategies have been used to increase the Cer levels, for instance the activation of SMase, which has demonstrated to be achieved by TNFα, etoposide or cytarabine (cytosine arabinoside—Ara-C) [83]; the epigenetic drug decitabine that demethylates SMase gene [1,6]; siRNA transfection; or analogues of glucosylceramide such as PDMP [52,84].

Table 1 shows some of the aforementioned agents and the different strategies used to target either the membrane directly or different steps in lipid metabolism pathways that ultimately result in changes in the membrane-lipid composition and biophysical status.

### 3.3. Lipid Replacement

The more rigid and less permeable membranes such it occurs in MDR cells creates an extra difficulty to the diffusion of chemotherapeutics. Since this barrier is essentially caused by increased cholesterol content and non-saturated/saturated lipid ratio, another strategy employed is the replacement of the lipids present in the membrane. Extensive experimental and clinical evidence suggests that the consumption of mono (oleic acid) and polyunsaturated fatty acids (PUFA), including docosahexaenoic acid (DHA), eicosapetaenoic acid (EPA) or linoleic acid contribute to prevent colon tumorigenesis. These fatty acids incorporate in the membrane phospholipids changing their composition, and, thus, its properties such as fluidity and phase behavior. Minerval, the synthetic fatty acid 2-hydroxyoleic acid (2OHOA) is a first-in-class in phase I/II clinical trials being tested for the treatment of glioma [85] (for an extended review see [4]). Also, propofol-DHA is being developed to treat breast cancer cells [7,84].

Apart of these changes in membrane properties, another bottleneck often encountered is the entrapment of chemotherapeutic drugs or protein-based therapeutics within endosomes upon uptake by cells. This may ultimately lead to drugs being released from cells after endosomes recycle back to the membrane or their degradation in lysosomes. Another class of molecules that were found to enhance the endosomal escape are saponins. Glycosylated triterpenoid saponins comprise a pentacyclic C30 perpene backbone structure with one or more covalently bound sugar chains [86]. Saponins have been used to enhance the cytotoxicity of several protein ligands in different cancer cell models (for a review see [87]).

## 4. The Anticancer Effects of the Bacterial Protein Azurin: A Cell Membrane Targeted Therapy

In the previous section, several molecules that can modulate the plasma membrane-lipid and/or protein contents and biophysical profile in cancer cells were presented. In this section, we present findings regarding a similar behavior of a bacterial protein acting at the membrane of cancer cells. Azurin is a small (14 kDa) membrane-associated protein from the *Pseudomonas aeruginosa* bacterium from the cupredoxin super family. In recent years, azurin has been intensively studied as an anticancer protein, down-regulating signaling pathways downstream of membrane receptors and functional processes such as adhesion and invasiveness [88,89,90,91,92,93,94].

### 4.1. Cancer Cells’ Membrane Modulation by Azurin

Recently, our group performed a microarray analysis in invasive breast cancer cells overexpressing P-cadherin, a cell-cell adhesion molecule that is aberrantly overexpressed in about 30% of breast cancers [95]. The results evidenced a very significant number of genes with altered expression coding for proteins associated with membrane related processes [93]. Endocytosis and the formation of early endosomes, vesicle mediated processes and membrane remodeling were some of the gene ontology and enriched pathways assigned by the Kegg database that were up-regulated by azurin. On the contrary, focal adhesion, cell motility, biological adhesion and cell surface receptors associated with signal transduction composed most of the genes down-regulated, assigned by the Kegg database [93]. The overexpression of P-cadherin leads to increased invasiveness and it has been associated with the aberrant expression of the α6β4 integrin dimmer which is also related to the hyperactivity of FAK, Src and AKT pro-tumorigenic and proliferative signaling [96]. Treating these cells with azurin led to a down-regulation of this cadherin protein levels, maintaining or even increasing E-cadherin levels, a known tumor suppressor cadherin protein [92]. Furthermore, at the functional level, invasion of the cells through Matrigel is severely impaired, in addition to the decrease of FAK and Src phosphorylation [92]. Indeed, treatments with azurin seem to have led to a global decrease of mechano-sensing associated pathways that drive the invasiveness in these models. Azurin also promoted a deficient adhesion of breast cancer cells to ECM proteins such as collagen, laminin and fibronectin, down-regulating the integrin subunits α6, β4 and β1 and decreases severely the mammosphere-forming efficiency of these cells [93].

In non-small cell lung cancer (NCSLC), azurin also decreases the expression of integrin subunit β1 as well as the adhesion to ECM proteins and the signaling pathways associated with increased proliferation such as PI3K/AKT and Src [88]. In this model, overexpression of this integrin is known to control the activity of EGFR. However, pre-treating the cells with azurin prevented the activation of EGFR pathway by its natural ligand EGF. Furthermore, the combination of azurin with EGFR-targeted therapies such as the small chemical inhibitors gefitinib or erlotinib produced a synergistic effect in the inhibition of proliferation stronger than with any of the chemicals alone [88].

At the membrane protein level, azurin also targets the receptor tyrosine kinase Eph receptor B2, competing with its natural ligand ephrin B2 for the binding to the receptor, therefore blocking the proliferative signaling induced by the natural ligand [92]. Later, engineered versions of peptides derived from azurin were conjugated to nicotinamide, increasing the sensitivity to radiotherapy up to 13-fold compared to the nicotinamide alone [93]. Using Surface Plasmon Resonance (SPR) to screen a library of peptides derived from the C-terminal region of azurin that would bind to ephrin receptors EphA2, EphB2 and EphB4, one particular analogue was found to sensitize Lewis lung carcinoma (LCC), both in vitro and in vivo, on solid tumor engraftment models [93]. These experiments increased the potential of this protein or derived peptides to target membrane-associated proteins relevant in different models of cancer.

The natural structure of azurin as an invariant β-sheet sandwich structure (Greek key β-barrel) formed by eight parallel and antiparallel strands and an extended α-helix region situated outside of the barrel, confers on this protein the nature of a scaffold protein. This also seems to give azurin the ability to interfere on multiple cancer–associated signaling events, exhibiting some promiscuity in its targets, unlike rationally designed drugs that target a specific step in a single pathway.

On top of the signaling and functional changes caused by azurin in the different cancer cell models, the cellular biophysical and nanomechanic properties of the plasma membranes were also evaluated. In lung cancer cells treated with azurin the membrane profile was assessed by Atomic Force Microscopy (AFM), in an attempt to explain the interference with cell attachment and response to growth factors [94]. The Young’s modulus (E) of azurin-treated lung cancer cells decreased upon treatment with azurin in about 30% [94]. This result seems to be in accordance with the increased sensitivity towards the EGFR kinase inhibitors, and as described in the previous sections, alterations in membrane stiffness in cancer cells often contribute to the reduced permeability to anticancer drugs. The global membrane order in different cancer cell models was also evaluated recently in three different cancer cell lines (MCF-7, A549 and HT-29) with the environment-sensitive probe Laurdan [97]. In this case, treating cells with azurin decreased the average GP value of plasma membranes, indicating a decrease in the Lo domains suggesting a more fluid membrane state.

Furthermore, a phenylalanine residue of azurin located in the surface hydrophobic patch of the protein is also involved in the entry into cancer cells, mediating interactions with caveolin-1 and GM-1, a ganglioside enriched in lipid rafts pointing to the importance of hydrophobic interactions between azurin and lipid raft resident lipids and proteins [97]. This also correlated well with increased sensitivity to paclitaxel and doxorubicin in the presence of azurin, again suggesting that a part of the anticancer effect of azurin may occur by changing the membrane properties in a way that favors the permeability to anticancer drugs and/or disturbs the pro-tumorigenic signaling of raft resident proteins.

### 4.2. Induction of Apoptosis

Further experiments also demonstrate that upon entry in cancer cells, azurin triggers apoptosis. Some evidence points to a possible interaction with p53, which would then support apoptosis [88,98,99,100,101]. A peptide derived from azurin, p28, is an amphipathic peptide that adopts an alpha-helical conformation with an overall negative net charge, is now the lead peptide in several pre-clinical and clinical studies towards different cancer types [102,103,104,105,106], based on its pro-apoptotic activity. The entry of this peptide seems to be dependent on the cholesterol levels present in the membrane since its depletion with MβCD strongly impaired its intracellular accumulation [107]. Several chemical inhibitors of different steps in endocytosis showed that p28 entered in cancer cells via caveosome-directed but also caveosome-independent pathways [107], and also that membrane bound glycosaminoglycans are apparently not involved in the uptake of azurin or p28 in the cells but N-glycosated proteins may play a role in the endocytosis of p28 [107]. The p28 peptide has also effects at the membrane since it blocks angiogenesis in endothelial and cancer cells through the modulation of the VEGFR-2 membrane receptor tyrosine kinase [108]. In endothelial HUVEC cells, p28 enters the cells co-localized with caveolin-1, blocking the VEGF and bFGF-induced migration, capillary tube formation and neoangiogenesis in several xenograft models. The down-regulation of VEGFR-2 induces the attenuation of FAK and AKT signaling pathways, namely through the decrease in their phosphorylation, which in turn, affects their signaling activity in the cellular remodeling associated with processes such as adhesion to the extracellular matrix and migration [108].

Other examples of protein-based anti-tumor therapies exist in the literature based on human proteins or peptides that act as cell-penetrating peptides (CPP) that also exert a pro-apoptotic effect in cancer cells. Lactoferrin (Lf) and its derived peptide lactoferricin (Lfcin) are extensively studied nutraceutical proteins with demonstrated activity against a broad spectrum um tumor types both as isolated agents as well as in combination with other chemotherapeutics such as tamoxifen [109]. In basal-like breast cancer, their combination resulted in enhanced apoptosis in mouse models of this breast cancer [110]. Another peptide derived from bovine lactoferricin with six amino acids, bLFcin_6_, demonstrated anti-tumor bioactivity and ability to deliver cargos, such as siRNAs, to the interior of cancer cells through a lipid raft-dependent micropinocytosis mechanism [111].

## 5. Conclusions and Future Perspectives

**Figure 2:**
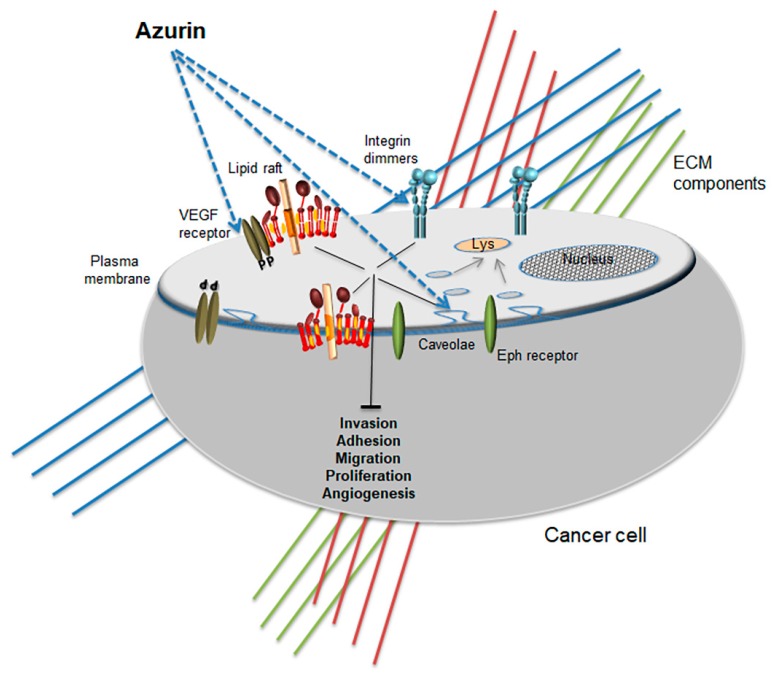
Promiscuous anticancer activity of azurin at the membrane level. Different membrane proteins are targeted by azurin, altering the biophysical profile and attenuating raft-related signaling pathways.

In conclusion, the role of the plasma membrane in cancer development and response to therapies is long known; however, only recently has it come to be consistently recognized as a target for the development of new therapies. Most cancer cells alter their lipid biosynthetic pathways in a way that not only favors proliferation and/or resistance to therapies but also induce significant changes in the biophysical properties of the plasma membrane. These alterations impact the interactions established with the vast majority of chemotherapeutics, small chemical compounds, and even biological therapies, protecting most of the times the cells of their activity. Different classes of drugs are therefore arising with the purpose of modulating the biochemical and biophysical features of cancer cells acting directly at the membrane and interacting with lipids and membrane-resident proteins or at the enzymes responsible for sensing and synthesis of membrane lipids. As proteins, azurin and its derived lead peptide p28, in this context, appear to be distinctive in their anticancer activity since through interactions with different plasma membrane raft components in cancer cells they can alter the biophysical profile and attenuate raft-related signaling pathways (Figure 2). The impact of azurin at cancer cells increases membrane fluidity and promotes endocytosis, thus providing opportunities to design new therapeutic strategies and drug-delivery systems based on their activity that may enhance the uptake and effectiveness of other drugs.

Their activity has been demonstrated in different models but it has not been demonstrated yet with more physiological models such as multidrug resistant cells or patient-derived cells, which would undoubtedly strengthen their role as new therapeutics or adjuvants to existent therapies in the most difficult-to-treat and resilient tumors that still represent a huge burden to human health.

## Figures and Tables

**Table 1:** Overview of selected therapeutic agents targeting multiple steps in lipid localization and synthesis to modulate membranes. Chemical structures were obtained from ChemSpider, a free chemical structure database owned by the Royal Society of Chemistry (http://www.chemspider.com/).

***Acting Directly at the Membrane***			**Ref**
*i) Lowering cholesterol levels*	Cholesterol binding agents: filipin	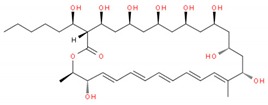	[6]
	Cholesterol chemical depletion: MβCD	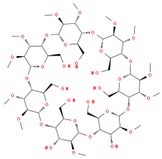	[38]
*ii) Stabilization of pro-apoptotic domains*	ALPs Edelfosine	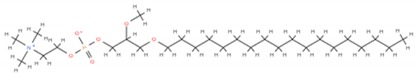	[70,71,72,73,74,75,76,77,78]
	Miltefosine	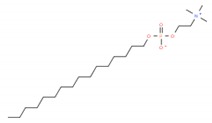	
	perifosine	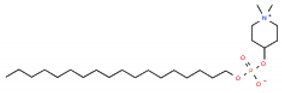	
	ODPC	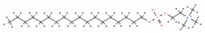	
***Acting Intracellularly in lipid metabolism pathways***			
*i) Fatty acid synthesis inhibition*	TVB-2640	unavailable	[62]
*ii) Statins (inhibition of mevalonate pathway)*	Lovastatin	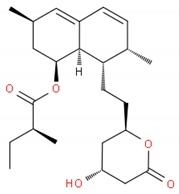	[65]
	Atorvastatin	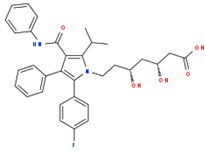	[66]
*iii) Cer metabolism (activation of SMase)*	Ara-C;	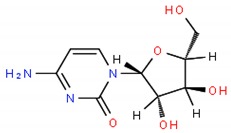	[83]
	Decitabine;	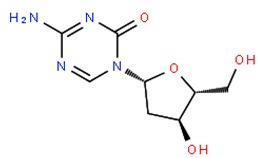	[1,6]
	glucosylceramide analogue (PDMP)	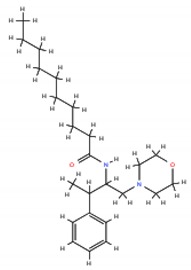	[52,84]
